# Anti-Inflammatory Activities of an Anti-Histamine Drug, Loratadine, by Suppressing TAK1 in AP-1 Pathway

**DOI:** 10.3390/ijms23073986

**Published:** 2022-04-03

**Authors:** Jiwon Jang, Stephanie Triseptya Hunto, Ji Won Kim, Hwa Pyoung Lee, Han Gyung Kim, Jae Youl Cho

**Affiliations:** 1 Department of Integrative Biotechnology, Sungkyunkwan University, Suwon 16419, Korea; rhea980327@gmail.com (J.J.); stephunto@gmail.com (S.T.H.); lauryun@naver.com (J.W.K.); leehwapyoung57@gmail.com (H.P.L.); 2 Biomedical Institute for Convergence at SKKU (BICS), Sungkyunkwan University, Suwon 16419, Korea

**Keywords:** Loratadine, anti-inflammatory effect, AP-1, TAK1

## Abstract

Loratadine is an anti-histamine routinely used for treating allergies. However, recent findings have shown that Loratadine may also have anti-inflammatory functions, while their exact mechanisms have not yet been fully uncovered. In this paper, we investigated whether Loratadine can be utilized as an anti-inflammatory drug through a series of in vitro and in vivo experiments using a murine macrophage cell line and an acute gastritis mouse model. Loratadine was found to dramatically reduce the expression of pro-inflammatory genes, including MMP1, MMP3, and MMP9, and inhibit AP-1 transcriptional activation, as demonstrated by the luciferase assay. Therefore, we decided to further explore its role in the AP-1 signaling pathway. The expression of c-Jun and c-Fos, AP-1 subunits, was repressed by Loratadine and, correspondingly, the expression of p-JNK, p-MKK7, and p-TAK1 was also inhibited. In addition, Loratadine was able to reduce gastric bleeding in acute gastritis-induced mice; Western blotting using the stomach samples showed reduced p-c-Fos protein levels. Loratadine was shown to effectively suppress inflammation by specifically targeting TAK1 and suppressing consequent AP-1 signaling pathway activation and inflammatory cytokine production.

## 1. Introduction

Inflammation is an immunological response to the intrusion of foreign substances into the body or tissue repair failure. It protects our bodies from such harmful external or internal stimuli by recruiting immune cells, which can attach to or eliminate foreign elements or injured tissue [1,2,3]. However, when the inflammatory response outstrips the level of infection, it becomes a critical factor in driving other diseases, including diabetes, arthritis, and cancer [4,5,6,7]. Immune cells express pattern recognition receptors (PRRs), which are responsible for recognizing damage/pathogen-associated molecular patterns (DAMPs/PAMPs) expressed by pathogens [8,9,10]. Some of the most widely studied mammalian PRRs are toll-like receptors (TLRs), which are responsible for inflammatory pathway activation. Among the 10 TLRs discovered in humans, TLR4 is responsible for lipopolysaccharide (LPS) recognition to identify representative disease-causing PAMPs expressed by gram-negative bacteria [11,12,13]. Once an LPS is bound to TLR4, sequential intracellular inflammatory signaling processes are initiated by myeloid differentiation factor 88 (MyD88) and TIR-domain-containing adaptor-inducing interferon-β (TRIF), two adaptor proteins attached to the cytosolic toll-interleukin-1 receptor (TIR) domain of TLR4. Nuclear factor-kappa B (NF-κB), interferon regulatory factor 3 (IRF3), and activator protein-1 (AP-1) signaling pathways are the three representative intracellular inflammatory pathways activated by MyD88 and TRIF [14,15,16].

Among them, the AP-1 pathway is regulated by the transcription factor AP-1, which is responsible for regulating cell differentiation, cell cycle progression, and apoptosis. The AP-1 pathway consists of c-Fos and c-Jun families, and its transcriptional activation is mediated through the dimerization of these two subunits [17,18,19,20,21]. Originally located in the cytosol, c-Fos and c-Jun translocate into the nucleus and dimerize after they are activated by their upstream kinases, mitogen-activated protein kinases (MAPKs). MAPKs comprise a family of serine/threonine protein kinases, notably including extracellular signal-regulated kinase (ERK), c-Jun N-terminal kinase (JNK), and p38. MAPKs are activated by upstream MAPK kinases (MAPKKs), such as MAPK/ERK kinase (MEK) and MAPK kinase (MKK). Activation of MAPKKs is mediated by MAPKK kinases (MAPKKKs), notably including transforming growth factor-β-activated kinase 1 (TAK-1), mixed lineage kinase 3 (MLK3), and apoptosis signal-regulating kinase 1 (ASK1) [22,23,24,25]. The activation of the above kinases is regulated by sequential phosphorylation from upstream kinases to downstream kinases. Activation of AP-1 allows the transcription of inflammation-related enzymes, including matrix metallopeptidases (MMPs) and cyclooxygenase 2 (COX-2) [26,27,28,29,30].

Histamine is a primary mediator of the allergic response. It is kept in mast cells or basophils until the cells receive certain stimuli allowing it to be released out of the cells [31,32]. When histamine interacts with histamine receptors H1, H2, H3, or H4, an allergic reaction occurs. Among them, drugs targeting the H1 receptor are most commonly used for the treatment of allergies [33,34]. Meanwhile, Loratadine is an H1 histamine receptor antagonist; therefore, it is widely used as allergy medication. More importantly, it is within the second generation of non-sedating anti-histamine drugs [35,36].

However, there have been several reports presenting the H1 receptor-independent anti-inflammatory effects of H1 receptor antagonists by inhibiting NF-κB and AP-1 activities. Fexofenadine, a widely-used anti-histamine drug, was also shown to have inhibitory effects with COX-2 in an H1-receptor-independent manner [37,38]. In the meantime, various studies have revealed that Loratadine may be used as a novel anti-inflammatory drug [39,40,41]. Similarly, our previous study of Loratadine showed that it possesses inflammation-suppressive activities in the NF-κB signaling pathway [42].

Nevertheless, its inflammation-regulatory activities in another important inflammatory response pathway, the AP-1 signaling pathway, remain undetermined. Therefore, in this paper, we investigate the exact mechanism of the anti-inflammatory effects of Loratadine in the AP-1 signaling pathway.

## 2. Results

### 2.1. Anti-Inflammatory Effects of Loratadine Are Mediated by Its Transcriptional Regulation of Pro-Inflammatory Genes

**Figure 1:**
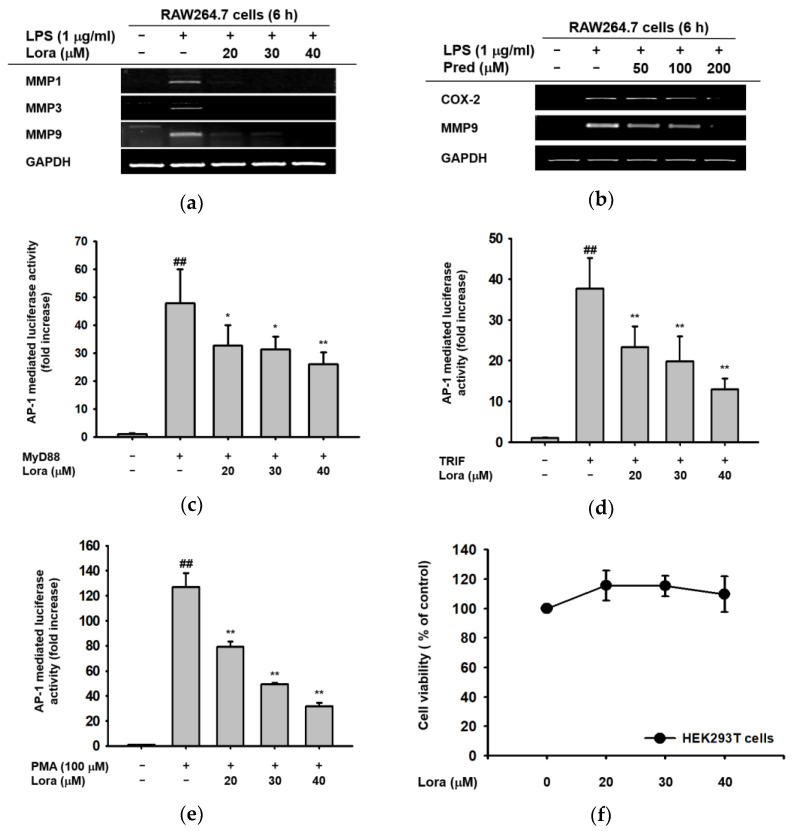
Inhibitory effects of Loratadine on the mRNA expression of pro-inflammatory genes and the transcriptional activation of inflammatory transcription factors. (**a**,**b**) RAW264.7 cells were pre-treated with the indicated concentrations of Loratadine (20–40 μM) or prednisolone (50–200 μM) for 30 min. The cells were treated with LPS (1 μg/mL) for 6 h, and the mRNA expression levels of MMP1, MMP3, MMP9, and GAPDH (control) were measured by reverse-transcription polymerase chain reaction (RT-PCR) and agarose gel electrophoresis. Visualization of DNA bands was achieved by exposing the gel to UV irradiation. (**c**–**f**) HEK293T cells were transfected with AP-1-luc, MyD88, TRIF, and β-galactosidase (control) using PEI for 24 h. Cells were treated with the indicated concentrations of Loratadine (20–40 μM) for 6 h before cell harvesting. The expression of AP-1 was measured by luciferase activity. (**f**) HEK293T cells were treated with the indicated doses of Loratadine (20–40 μM) for 24 h, and viability was measured using the MTT cell viability assay. ## *p* < 0.01 compared to normal group; * *p* < 0.05 and ** *p* < 0.01 compared to control group. All data are presented (**a**–**f**) as mean ± standard deviation of the experiments performed with 4–6 samples.

First, in order to determine whether or not Loratadine has anti-inflammatory effects on the transcriptional level, we examined the mRNA expression levels of pro-inflammatory genes as well as the activation of transcription factor AP-1 under inflammation-triggered conditions. In RAW264.7 cells treated with LPS (1 μM), Loratadine (20–40 μM) effectively suppressed the mRNA expression of MMP1, MMP3, and MMP9 (Figure 1a), which are representative pro-inflammatory factors expressed via AP-1 pathway activation [43,44]. In addition, for further rationalization of such an effect, we also conducted the same experiment with prednisolone, an FDA-proved anti-allergic and anti-inflammatory drug [45], as a positive control. As shown in Figure 1b, prednisolone also suppressed the expression of COX-2 and MMP9 in a dose-dependent manner, supporting the validity of Loratadine’s anti-inflammatory effects indicated by MMP-reducing behavior. Therefore, we decided to further investigate whether such reduction in pro-inflammatory gene expression was a consequence of direct AP-1 inhibition by Loratadine.

In MyD88- and TRIF-overexpressed or PMA (100 μM)-treated HEK293T cells, AP-1-luc activation levels were significantly reduced in a concentration-dependent manner by Loratadine treatment (20–40 μM), as indicated in Figure 1c–e. The figures clearly indicate that AP-1 promoter activity, in other words, AP-1 transcription, is suppressed by Loratadine treatment. Furthermore, the cell viability assay showed that Loratadine treatment (20–40 μM) did not have any cytotoxicity at investigated concentrations (Figure 1f), implying that the reduction in AP-1 transcription was not due to cell death. Therefore, we decided to set the target concentration of Loratadine as 20–40 μM since these three concentrations showed a dose-dependent mode of action, and none of them showed cell toxicity. In conclusion, these data suggest that Loratadine suppresses pro-inflammatory factors via transcriptional regulation, and such effects are not outcomes of cell death.

### 2.2. Loratadine Targets the AP-1 Signaling Pathway to Reduce Inflammatory Responses Both In Vitro and In Vivo

**Figure 2:**
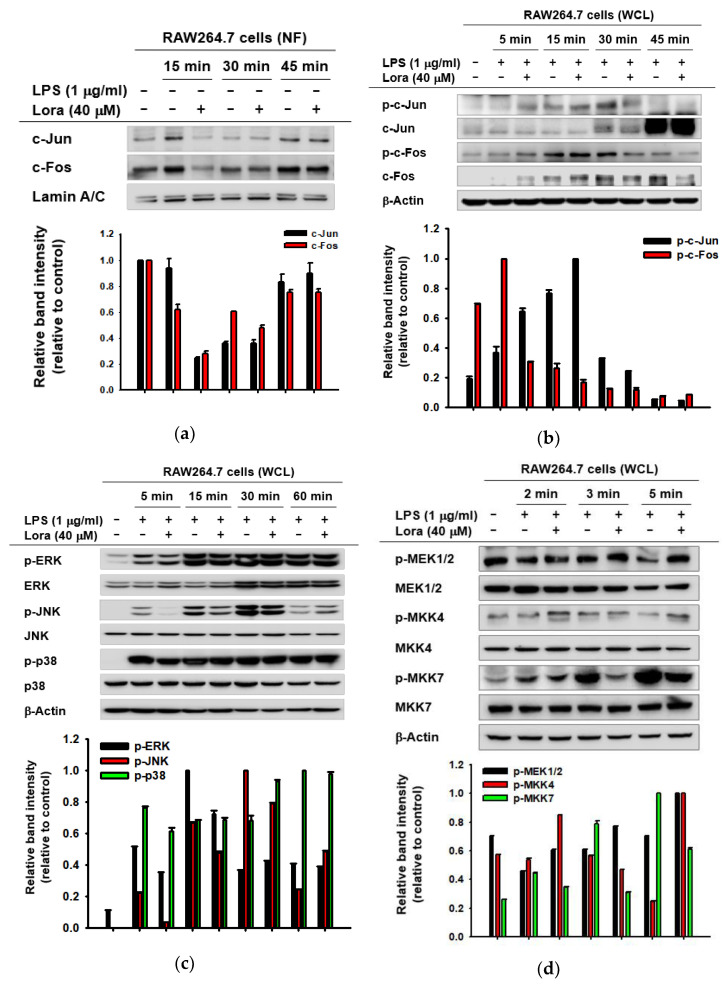

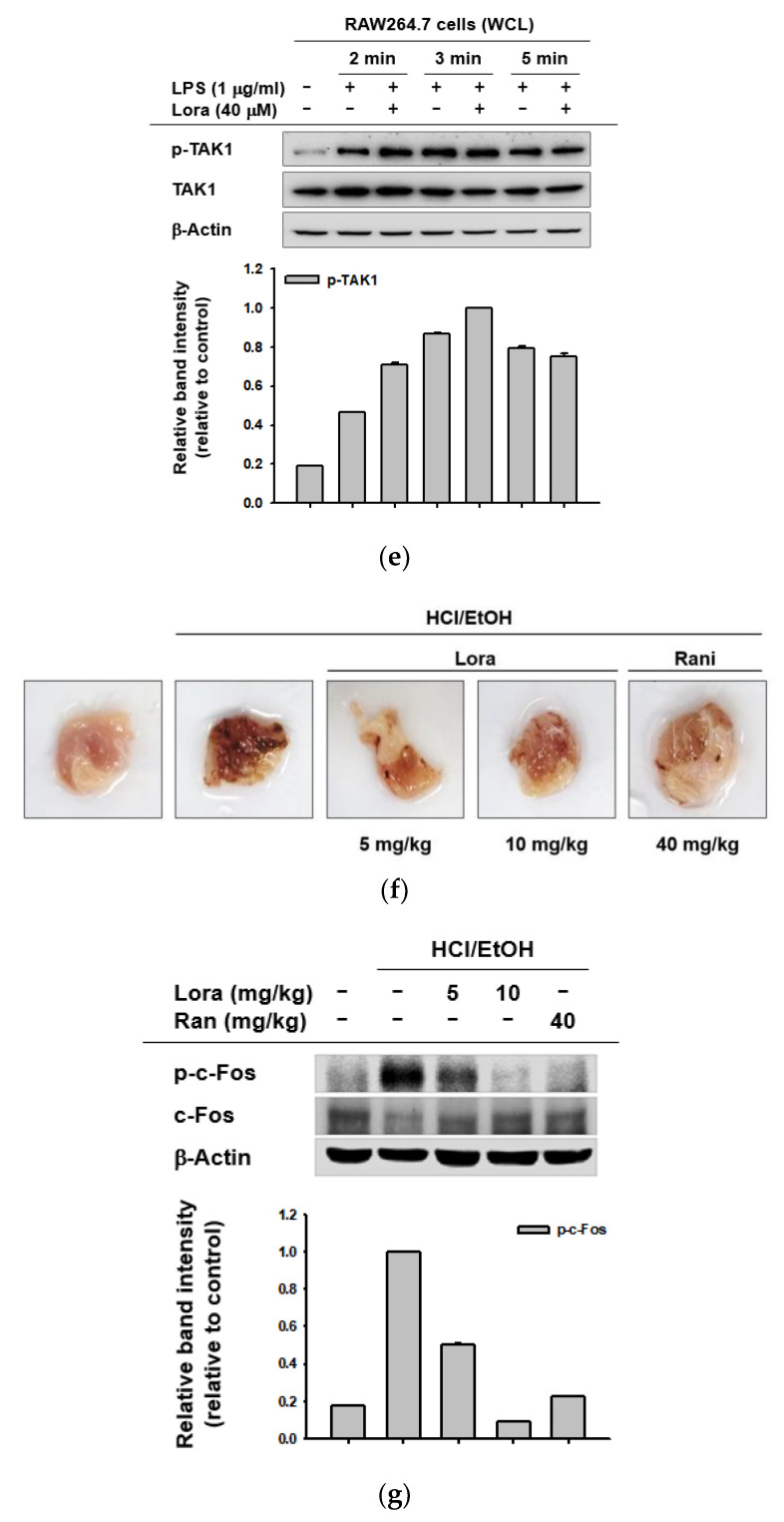
Anti-inflammatory effects of Loratadine on the constituent kinases of the AP-1 signaling pathway. (**a**) RAW264.7 cells were pre-treated with Loratadine (40 μM) for 30 min following LPS treatment (1 μg/mL) for the indicated time points (15–45 min). The expression levels of c-Jun, c-Fos, and Lamin A/C (control) within the nuclear fraction were assessed by Western blotting analysis. (**b**–**e**) RAW264.7 cells were pre-treated with Loratadine (40 μM) for 30 min following LPS treatment (1 μg/mL) for the indicated time points (5–60 min). The levels of total or phosphorylated c-Jun, c-Fos, ERK, JNK, p-38, MEK1/2, MKK4, MKK7, TAK-1, and β-actin (control) within the whole lysate were measured by Western blotting analysis. NF: nuclear fraction; WCL: whole cell lysate. (**f**) Acute gastritis was induced using HCl/EtOH after vehicle, Loratadine, or ranitidine injection. Stomach samples were obtained and cut open after sacrifice to assess stomach bleeding levels. (**g**) Stomach samples were ground in liquid nitrogen and lysed using cell lysis buffer. Western blotting was performed to assess the expression levels of total and phosphorylated c-Fos and β-Actin (control). NF: nuclear fraction; WCL: whole cell lysate; Ran: Ranitidine. (Bottom panels of **a**–**e**,**g**). Relative intensity of these proteins was calculated by ImageJ.

Since we discovered suppressive effects of Loratadine on AP-1-mediated gene expression and promoter activity of AP-1 (Figure 1), we further investigated whether such anti-inflammatory effects could also affect the constituent molecules of the intracellular AP-1 signaling pathway. Based on the concept that two subunits of AP-1, c-Jun, and c-Fos, translocate from the cytosol to the nucleus upon activation [46], the expression levels of c-Jun and c-Fos were determined within the nuclear fraction. In RAW264.7 cells, the nuclear expression levels of both c-Jun and c-Fos were clearly decreased following Loratadine treatment (40 μM) 15 min after LPS induction (Figure 2a). On the other hand, total cell lysate also showed decreased phosphorylation levels of c-Jun at 30 min and c-Fos at 30 and 45 min after LPS treatment (Figure 2b). It clearly indicates that Loratadine inhibits the activity of c-Jun and c-Fos. In addition, decreased levels of total c-Jun and c-Fos by Loratadine imply that Loratadine may also be involved in the degradation of the transcription factors.

Subsequently, we evaluated the cytosolic levels of AP-1 upstream kinases in LPS-induced RAW264.7 cells. Among the three different MAPKs, ERK, JNK, and p38, p-JNK (phosphorylated JNK) levels were clearly decreased 5 and 15 min post-inflammation induction by Loratadine treatment (40 μM), as shown in Figure 2c. Consistently, we also found the expression levels of p-MKK7 (phosphorylated MKK7), an upstream molecule of JNK, to be decreased by the same concentration of Loratadine 3 and 5 min post-induction (Figure 2d). Other MAPKKs, including MEK1/2 and MKK4, did not show such a clear reduction (Figure 2d). Finally, the expression levels of TAK1, which is a member of the MAPKKK family and an upstream activator of MKK7, were examined. LPS induction and Loratadine treatment (40 μM) were not found to reduce p-TAK1 (phosphorylated TAK1) levels (Figure 2e). Under inflammation-triggering conditions, a reduction in molecules downstream from TAK1 but not p-TAK1 itself indicates that TAK1, among other molecules of the AP-1 signaling pathway, might be the primary target of Loratadine.

In addition, in order to demonstrate the anti-inflammatory effects of Loratadine in vivo, we investigated a hydrochloride (HCl)-induced acute gastritis mouse model. Ethanol-induced gastritis leads to increased gastric acid secretion, which results in sequential activation of c-AMP, H2R, and H+/K+ ATPase, eventually causing gastric mucosal damage [47,48,49]. On the other hand, Ranitidine is known to be a potential H2R antagonist, inhibiting H2R-mediated gastric damage [50,51]. Therefore, we decided to use Ranitidine as a control drug in the experiment. HCl/EtOH-induced mice showed the typical phenotype of acute gastritis, bleeding of the stomach. However, Loratadine (5, 10 mg/kg) injection significantly reduced gastric bleeding, and its effect was comparable to Ranitidine (40 mg/kg) (Figure 2f). Moreover, phosphorylated c-Fos levels were dose-dependently decreased in stomach samples from Loratadine-treated mice compared to those of the vehicle-treated group (Figure 2g). These results suggest that the anti-inflammatory effects of Loratadine are mediated by its suppressive activity in the AP-1 signaling pathway.

### 2.3. TAK1 Is the Prime Target Molecule of Loratadine

**Figure 3:**
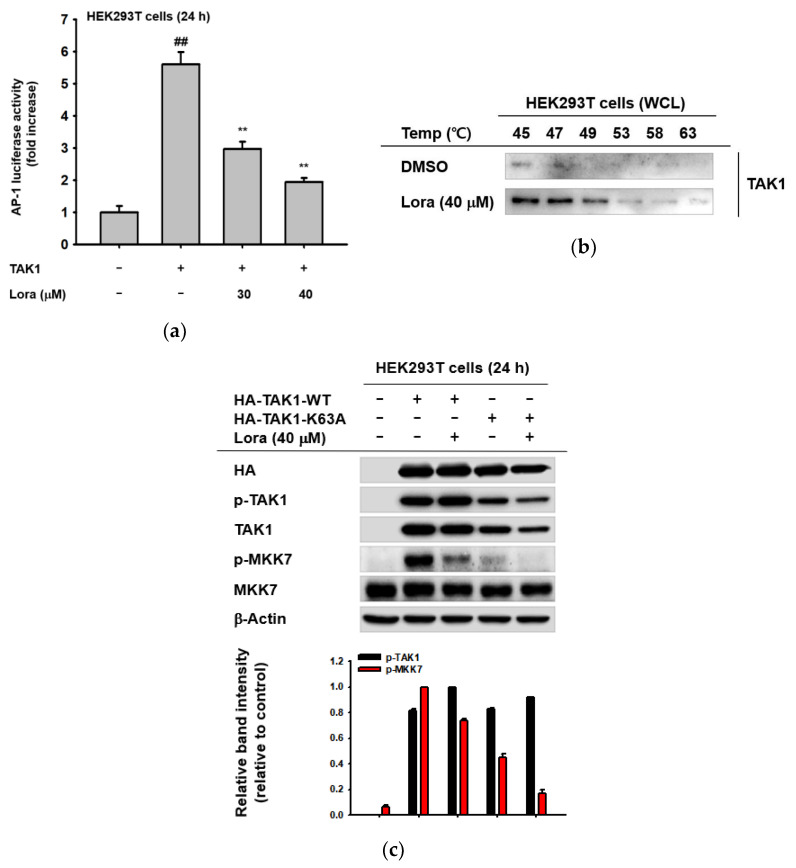
Loratadine specifically targets TAK-1 to exert anti-inflammatory activity. (**a**) HEK293T cells were transfected with AP-1-luc, HA-TAK-1, and β-galactosidase (control) using PEI for 24 h. Cells were treated with the indicated doses of Loratadine (30–40 μM) for 1 h. The expression level of AP-1 was assessed by measuring luciferase activity. (**b**) HEK293T cells were transfected with TAK1 using PEI for 24 h and treated with Loratadine (40 μM) for 1 h after transfection. The binding affinity of TAK-1 and Loratadine was evaluated using the cellular thermal shift assay at the indicated temperatures. (**c**) HEK293T cells were transfected with HA-TAK-1-WT or HA-TAK-1-K63A constructs using PEI for 24 h and treated with Loratadine (40 μM) for 1 h and whole-cell lysates were prepared. The expression levels of total or phosphorylated TAK-1, MKK7, HA, and β-actin (control) were measured through Western blotting analysis. (Bottom panel of **c**) Relative intensity of these proteins was calculated by ImageJ. ## *p* < 0.01 compared to the normal group; ** *p* < 0.01 compared to the control group. Data are presented as mean ± the standard deviation of experiments performed with 4–6 samples. WCL: whole cell lysate.

In order to determine whether TAK1 is the primary target of Loratadine, we further examined the drug’s anti-inflammatory activity in TAK1-overexpressing cells. Overexpression of TAK1 can induce and activate its downstream molecules, resulting in levels of inflammation similar to cells treated with LPS [52,53]. First, we decided to check the expression of pro-inflammatory cytokines after TAK1 overexpression. When TAK1 was overexpressed in RAW264.7 cells, COX-2 expression was significantly reduced following Loratadine treatment (40 μM) (data not shown). Furthermore, TAK1 overexpression in HEK293T cells induced AP-1 promoter activation; however, it was dose-dependently suppressed by Loratadine (30 and 40 μM) (Figure 3a). These results suggest that TAK1-induced inflammation can be suppressed by Loratadine treatment, implying that Loratadine may directly interact with TAK1. Therefore, we performed the cellular thermal shift assay (CETSA) to find out whether or not Loratadine directly targets and binds TAK1 to suppress the AP-1 signaling pathway during inflammation. Compared to the unbound state, the thermal stability of TAK1 is higher when it is bound with other molecules. Within 45 to 63 degrees Celsius, the TAK1 band intensity of DMSO-treated cells was clearly lower than that of Loratadine (40 μM)-treated cells, showing a reducing pattern as the temperature rose (Figure 3b). This indicates that Loratadine and TAK1 interact and may directly bind. In addition, we decided to further investigate the target binding site of TAK1 for Loratadine. TAK1 has an ATP binding site, K63, which enables it to transform into an active state upon ATP binding [54,55]. In order to find out whether K63 of TAK1 is the target binding site for Loratadine, we point-mutated K63 into A63. If K63 was the binding site, mutated TAK1 with A63 would show no change in expression of both p-TAK1 and p-MKK7. However, we found that there was still a reducing pattern of p-MKK7 in K63A TAK1-transfected HEK293T cells after Loratadine treatment (40 μM) (Figure 3c), indicating that Loratadine does not appear to bind to K63. These results suggest that Loratadine targets TAK1 within the AP-1 signaling pathway to exert anti-inflammatory effects, and the ATP-binding site of TAK1 is not the target site for Loratadine.

## 3. Discussion

Inflammation is a fundamental process for maintaining immunologic homeostasis; however, an excessive inflammatory response can lead to severe disorders, including autoimmune diseases and cancer [56,57,58]. In this paper, we demonstrate the anti-inflammatory activity of a commercialized anti-histamine drug, Loratadine, which specifically inhibits TAK1 activation and, therefore, suppresses the AP-1 signaling pathway and consequent MMP production. We have previously reported that Loratadine can reduce several pro-inflammatory cytokines, including iNOS, IL-1β, TNF-α, IL-6, and COX-2, by suppressing the NF-κB signaling pathway [42]. These findings suggest that Loratadine is able to counteract exaggerated inflammatory responses, thereby maintaining immunological balance while preventing the uncontrolled progression of inflammation.

Recently, drugs targeting the AP-1 pathway have emerged as next-generation treatments for both cancer and autoimmune disease. The AP-1 pathway governs major inflammatory responses by regulating cell differentiation, proliferation, apoptosis, angiogenesis, and invasion [59,60,61,62,63]. Meanwhile, AP-1 is composed of different combinations of the Fos and Jun families. The Fos family includes c-Fos, FosB, and Fra-1 and -2, whereas the Jun family includes c-Jun, JunB, and JunD [61,64,65]. Each of them controls different cellular responses by up- or down-regulating related gene products. C-Fos and c-Jun up-regulate factors that are responsible for proliferation, such as EGFR, GM-CSF, and cyclin D1 [66,67,68,69]. On the other hand, c-Jun also down-regulates proteins that inhibit proliferation and stimulate apoptosis, such as p53 and FASL [70,71,72,73]. In addition, c-Fos can up-regulate genes that promote cellular invasions, such as MMP1, MMP3, and CD44 [74,75,76,77]. Therefore, AP-1 has been a foremost target for cancer and inflammatory disease treatment. For instance, T-5224, a selective inhibitor of c-Fos/AP-1, has been reported to have preventive effects on cartilage destruction and osteophyte formation [78]. Furthermore, a selective small-molecule inhibitor of c-Fos/AP-1 has been shown to resolve arthritis in pre-clinical models [79].

TAK1, a constituent molecule of the AP-1 signaling pathway, is also a chief regulator of cell survival and apoptosis, which has made itself a promising target for a wide set of diseases. For instance, Takinib, a TAK1-specific inhibitor, was reported to mediate cell death in rheumatoid arthritis and breast cancer by suppressing TNF-α production [80,81]. Other TAK1 inhibitors, such as LYTAK1 and (5Z)-7-Oxozeaenol, also showed suppressive effects on various types of cancer, including ovarian cancer, cervical cancer, and neuroblastoma [82,83,84,85].

Loratadine is one of the most frequently used anti-allergic drugs, whose safety and efficacy have already been approved by the FDA. Together with the findings that outline AP-1 and TAK1 as being promising drug targets, our paper suggests the potential of Loratadine as an operative anti-inflammatory agent, with known and manageable side effects and proven safety and stability. Furthermore, such findings will also contribute to future research on drug repositioning by providing proper methods and workflows for discovering new effects of pre-existing commercial drugs on different diseases.

## 4. Materials and Methods

### 4.1. Materials

Loratadine was purchased from Tokyo Chemical Industry Co., LTD. (Tokyo, Japan). Carboxymethylcellulose (CMC), dimethylsulfoxide (DMSO), L-NAME, lipopolysaccharide, 3-(4,5-dimethylthiazol-2yl)-2,5-diphenyltetrazolium bromide (MTT), polyethyleneimine (PEI), Ranitidine, and stillen were obtained from Sigma Aldrich (St. Louis, MO, USA). DMEM, fetal bovine serum (FBS), penicillin/streptomycin, phosphate-buffered saline (PBS), RPMI 1640, trypsin, and TRIzol were acquired from GIBCO (Grand Island, NY, USA). HEK293T (CRL-1573) and RAW264.7 cells (CRL-2278) were products from American Type Culture Collection (Rockville, MD, USA). Luciferase constructs with AP-1 binding sites and CFP-TRIF, FLAG-MYD88, pcDNA, and Tag-2 constructs were used as previously reported [86]. Real-time and semiquantitative reverse transcriptase-polymerase chain reaction primers were bought from Macrogen Inc. (Seoul, Korea). All other chemicals were purchased from Sigma. Total and phospho antibodies for the MAPK family were obtained from Cell Signaling Technology (Beverly, MA, USA) and Santa Cruz Biotechnology (Santa Cruz, CA, USA).

### 4.2. Compound Preparation, Animals, and Cell Culture

First, 40 mM of Loratadine stock solution was prepared using DMSO as a carrier. The stock solution was further diluted and treated at final concentrations of 20–40 μM using cell culture media for in vitro experiments. For in vivo procedures, 5–10 mg/kg using 0.5% sodium carboxymethyl cellulose (CMC) solution as a carrier. Male ICR mice (5–6 weeks old) were purchased from Daehan Biolink (Osong, Korea) and were housed in autoclaved plastic cages under standard housing conditions. All studies were conducted according to the guidelines of the Institutional Animal Care and Use Committee at Sungkyunkwan University (Suwon, Korea; approval ID: 2019-12-03-1). Murine macrophage RAW264.7 cells and human embryonic kidney HEK293T cells were cultured in RPMI1640 and DMEM media, respectively, containing 5 or 10% inactivated FBS, penicillin (100 IU/mL), and streptomycin (100 μg/mL). Cells were cultured in an incubator that maintained a 5% CO_2_ level and a temperature of 37 °C [87,88].

### 4.3. Gastritis Mouse Model

Negative and positive control groups, 5 ICR mice per group, received oral administration of 0.5% CMC solution, while the two Loratadine-treated groups were orally injected with 5 mg/kg or 10 mg/kg Loratadine solution. The control group was injected with 40 mg/kg Ranitidine. The injection was performed twice a day for 3 days. For acute gastritis induction, mice received orally administered 150 mM hydrochloric acid (HCl) in 60% ethanol (EtOH) 1 h before sacrifice. The stomach of each mouse was extracted to measure the area of inflammatory lesions. Quantification of the lesion area was performed using ImageJ [88].

### 4.4. mRNA Expression Analysis Using Reverse-Transcription Polymerase Chain Reaction

RAW264.7 cells (1 × 10^6^ cells/mL) were seeded in 12-well culture plates and incubated for 24 h. Loratadine (20–40 μM) or DMSO (as a control) was administered to cells 30 min before LPS induction (1 μg/mL). After 6 h of incubation, cells were harvested using cold PBS, and total RNA was isolated from the cells using TRIzol reagent following the manufacturer’s guidelines. In order to increase sample stability, cDNA was synthesized from the extracted mRNA using Thermo Fisher Scientific’s cDNA synthesis kit (Waltham, MA, USA); corresponding primers of COX-2, MMP1, MMP3, and MMP9 were applied for general PCR amplification. mRNA expressions of samples were analyzed by agarose gel electrophoresis; PCR band intensities were quantified using ImageJ. Primer sequences are listed in Table 1 [89].

### 4.5. Luciferase Reporter Assay

HEK293T cells (1.2 × 10^5^ cells/mL) were seeded in 24-well culture plates and incubated for 24 h. Target gene constructs (Tag2-MyD88, Tag2, CFP-TRIF, CFP, HA-TAK1, HA, AP-1-luc, and β-gal) were then transfected into the cells using PEI. After 24, 40, or 48 h incubation, the original culture media was completely removed, and Loratadine (20–40 μM) or DMSO were introduced to the cells with fresh media. After incubating for 3 h, the media was completely removed, and the cells were treated with luciferase lysis buffer. The plate was frozen at −70 °C for 3 h. Luciferin and β-gal assay buffers were added for luciferase and β-gal analysis, respectively. Luciferase activity was assessed by detecting luminescence levels, and β-gal activity was measured through OD values at 405 nm [90].

### 4.6. Cell Viability Assay

HEK293T cells (1 × 10^5^ cells/mL) were seeded in 96-well culture plates and incubated for 24 h. Loratadine (20–40 μM) or DMSO (as a control) were administered, and the cells were incubated for 24 h. MTT solution was added to the cell-containing medium, which underwent incubation for 3 h. MTT stopping solution was added thereafter. After 24 h of incubation, the cell viability was assessed through OD values at 570 nm [91].

### 4.7. Total Cell Lysate Preparation

RAW264.7 cells (2.5 × 10^6^ cells/mL) were seeded in 3 cm culture plates and incubated for 24 h. The cells were pre-treated with Loratadine (20–40 μM) or DMSO (as a control) for 30 min prior to inflammation induction. The cells were then treated with LPS (1 μg/mL) for specific periods (2–60 min), and the cells were then harvested using cold PBS. Total cell lysates were prepared using cold cell lysis buffer (20 mM of Tris-HCl, pH: 7.4; including 2 mM of EDTA, 2 mM of EGTA, 50 mM glycerol phosphate, 1 mM of DTT, 2 μg/mL of aprotinin, 2 μg/mL of leupeptin, 1 μg/mL of pepstatin, 50 μM of PMSF, 1 mM of benzamide, 2% Triton X-100, 10% glycerol, 0.1 mM of sodium vanadate, 1.6 mM of pervanadate, and 20 mM of NaF). Cell debris was removed by centrifugation at 12,000 RPM for 1 min. Total cell lysates were kept at −70 °C until use [87,92].

### 4.8. Western Blotting

Protein concentrations of total cell lysate samples were quantified for size-dependent separation. Proteins were separated by SDS-polyacrylamide gel electrophoresis in SDS-PAGE running buffer (10% SDS, Tris-base, glycine). Subsequently, proteins were transferred to PVDF membranes by electrophoresis in a transfer buffer solution (10% SDS, Tris-base, glycine, methanol). Membrane blocking was performed by rolling the tubes on a roller with 5% BSA solution applied for 1 h at room temperature. Primary antibodies specific to the target proteins (total or phosphorylated c-Jun, c-Fos, Lamin A/C, ERK, JNK, p38, MEK1/2, MKK4, MKK7, ASK, MLK3, TAK-1, and β-actin) were added, and the solutions were incubated with 5% BSA solution for 1–2 h at room temperature or overnight at 4 °C. After washing primary antibodies 3 times with 0.1% TBST buffer (Tris-base, NaCl; 0.1% Tween 20; pH: 7.6), specific HRP-linked secondary antibodies (anti-rabbit or anti-mouse) were added, and the solutions were incubated with 5% BSA solution for 1–2 h at room temperature or overnight at 4 °C. ECL reagent was applied for protein visualization, and signals were detected by ChemiDoc XRS (Bio-Rad, Hercules, CA, USA.). The relative intensity of each band was analyzed through the ImageJ program [93].

### 4.9. Gene Overexpression

HEK293T cells (2 × 10^5^ cells/mL) were seeded in 12-well culture plates and incubated for 24 h. Target gene construct and its carrier vector (HA-TAK1 and HA) were then transfected into cells using PEI. After 24 h, the original media was completely removed, and Loratadine (40 μM) was introduced to the cells in addition to fresh media. After further incubation for 3 h, media containing Loratadine was completely removed, the cells were harvested, and the total cell lysates were prepared. Protein expression levels were assessed by Western blotting using ECL reagents [94].

### 4.10. Cellular Thermal Shift Assay

HEK293T cells (1.2 × 10^6^ cells/mL) were seeded in 6 cm culture plates and incubated for 24 h. The target gene construct (HA-TAK1) was transfected into cells using PEI. After 24 h of incubation, Loratadine (40 μM) was introduced to the cells after the original media was replaced by fresh media, and the cells were thereafter incubated for 1 h. After 24 h of incubation, the cells were counted, and each group was adjusted to have an identical number of cells. Cells were lysed in a real-time PCR machine (Bio-Rad) within a temperature range of 45 to 63 °C. After a temperature shift, the protein level of TAK1 was analyzed by Western blotting [95].

## 5. Conclusions

**Figure 4:**
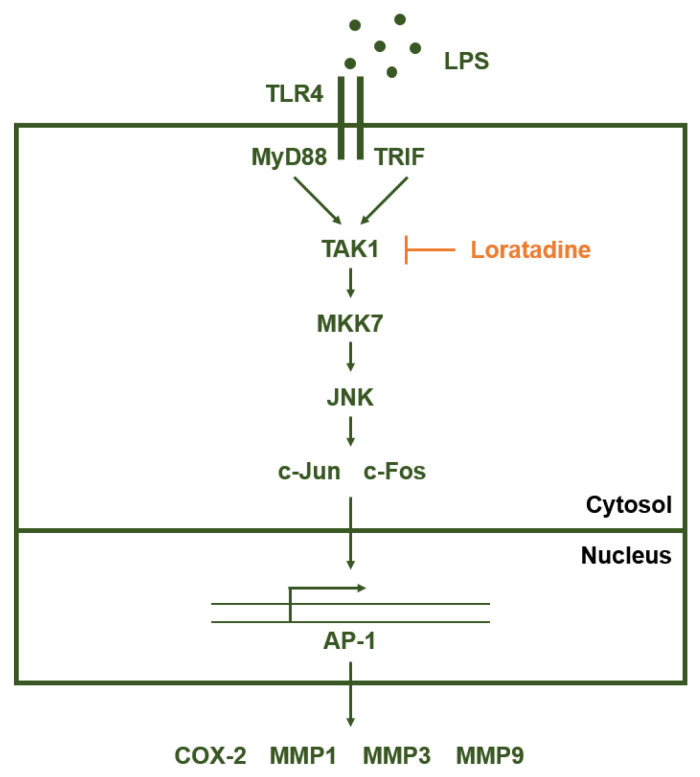
Schematic representation of Loratadine targeting the AP-1 signaling pathway. Loratadine specifically inhibits TAK1 in the AP-1 signaling pathway upon LPS induction, thereby suppressing the constituent kinases of the AP-1 pathway and eventually reducing the production of pro-inflammatory cytokines.

Loratadine exhibits anti-inflammatory activity in murine macrophage cells by specifically inhibiting the AP-1 signaling pathway. During this process, Loratadine targets TAK1 to suppress AP-1 transcriptional activity, thereby reducing pro-inflammatory cytokine expression, including that of MMP1, MMP3, and MMP9. Loratadine can also exert such inflammation-suppressing effects in an acute gastritis mouse model, implying its potential as an effective anti-inflammatory drug (Figure 4).

## Figures and Tables

**Table 1:** Primer sequences used in this study.

Gene Name		Sequence (5′ to 3′)
MMP-1	F	GCCTGCGTCCATCAACACT
	R	CCCTCCTCGTCCACCTCAA
MMP-3	F	ACTCCCTGGGACTCTACCAC
	R	TTCTTCACGGTTGCAGGGAG
MMP-9	F	TCTTCCCCAAAGACCTGAAA
	R	TGATGTTATGATGGTCCCAC
COX-2	F	GGGAGTCTGGAACATTGTGAA
	R	GCACATTGTAAGTAGGTGGACTGT
GAPDH	F	CAATGAATACGGCRACAGCA
	R	AGGGAGATGCTGGTTGG

F: Forward, R: Reverse.

## Data Availability

The data used to support the findings of this study are available from the corresponding authors upon request.
